# Comparison between kinesiotherapy and usual care during the first stage of labour in misoprostol-induced pregnant women: randomised clinical trial

**DOI:** 10.61622/rbgo/2025rbgo95

**Published:** 2025-11-18

**Authors:** Milene de Oliveira Almeida, Alexandre Delgado, Rita de Cassia Barros da Silva, Fernanda César Alves, Nadine Oliveira Cabral, Monique Maria Silva da Paz, Maryllian de Albuquerque Vieira, Thais Josy Castro Freire de Assis, Andrea Lemos

**Affiliations:** 1 Universidade Federal de Pernambuco Recife PE Brazil Universidade Federal de Pernambuco, Recife, PE, Brazil.; 2 Instituto de Medicina Integral Prof. Fernando Figueira Recife PE Brazil Instituto de Medicina Integral Prof. Fernando Figueira, Recife, PE, Brazil.; 3 Universidade Federal da Paraíba João Pessoa PB Brazil Universidade Federal da Paraíba, João Pessoa, PB, Brazil.; 4 Universidade de Brasília Brasília DF Brazil Universidade de Brasília, Brasília, DF, Brazil.; 5 Universidade Federal de São Carlos São Carlos SP Brazil Universidade Federal de São Carlos, São Carlos, SP, Brazil.

**Keywords:** Pregnant woman, Misoprostol, Applied kinesiology, Obstetric labour, Delivery, obstetric, Physical therapy modalities, Excercise

## Abstract

**Objective:**

To analyse the effectiveness of kinesiotherapy in the labour of pregnant women induced by misoprostol.

**Methods:**

This is a clinical trial carried out with pregnant women pharmacologically induced by misoprostol (25mcg, vaginally). They were randomised into two groups: Intervention Group (IG) of induced pregnant women who underwent kinesiotherapy during the active phase of labour; and Control Group (CG), of induced pregnant women who did not do physical therapy during the active phase of labour. Among the variables studied were vaginal delivery, induction time, duration of the labour, and the number of misoprostol doses. Data were analysed by the Statistical Package for the Social Sciences (SPSS). The IG had more vaginal deliveries (p=0.016). The other variables analysed showed no difference between groups.

**Results:**

Kinesiotherapy during the active phase of labour in women induced by misoprostol was effective for performing more vaginal deliveries despite not showing any difference in the other outcomes studied.

**Conclusion:**

The findings of this study indicate that after the application of kinesiotherapy, during the active phase of labour in pregnant women induced by misoprostol, the number of vaginal deliveries increases.

**Brazilian Registry of Clinical Trials (REBEC):**

RBR-3mvj4m7

## Introduction

Labor is divided into three stages, each with distinct durations and physiological characteristics. The first stage extends from the onset of regular uterine contractions to complete cervical dilatation (10 cm). It is subdivided into two phases: the latent phase, characterized by cervical dilatation up to 5 cm, and the active phase, during which dilatation progresses from 5 cm to 10 cm.^(1)^ The induction of labour aims to stimulate cervical ripening and provide the beginning of uterine contractions in those cases where this process did not start spontaneously, producing labour in a more real way and favouring a vaginal delivery.^(2)^

One of the forms of pharmacological induction that has been extensively researched and used in obstetric practice is misoprostol, a synthetic prostaglandin E1 analogue.^(3)^ For being a stable drug at room temperature and of low commercial value, misoprostol has gained popularity as an inducing agent in recent years. Current World Health Organization (WHO) guidelines recommend that, for vaginal administration, a dose of 25 mg of misoprostol should be administered every 6 hours.^(4)^ Misoprostol promotes cervical ripening by inducing collagen breakdown in the stromal tissue and stimulates uterine contractions through its binding to smooth muscle cells in the uterus.^(3)^ The literature shows that even after the induction process, a good part of pregnant women (31 to 44.3%) still progress to caesarean section.^(5,6)^ It was also observed that the main indications described for surgical intervention were a failure in induction and non-reassuring fetal status.^(5,6)^

The caesarean delivery route continues to grow around the world. According to a recent survey by the World Health Organization (WHO), about 21.1% of women delivered by caesarean section in recent years, a percentage that can reach up to 42.8% in some regions of the world.^(7)^ According to the WHO guidelines, the ideal caesarean rate should not exceed 15%.

Interventions that allow verticalization and maternal mobility can act positively in labour, as these postures contribute to a reduction in labour time and the number of caesarean sections.^(8)^

The physical therapist has techniques that help the parturient to remain active during this period, such as kinesiotherapy, which allows her to adopt different vertical postures such as standing, sitting, kneeling, and squatting, providing a more effective uterine contraction.^(5)^ Kinesiotherapy is a therapy that uses movement and applies principles of anatomy, physiology and biomechanics, promoting better maternal positioning during labor.^(9)^

By adopting vertical postures during labour, it is possible to decrease the duration of the second stage of labour, as well as the rates of episiotomy. Postures such as the squat favour an increase in pelvic diameters in the anteroposterior and transverse directions of up to 6.1 mm and 11.0 mm respectively.^(10)^

The Swiss ball is a low-cost resource that can be used by the professional during labour, where it promotes a decrease in the level of maternal pain and consequently helps in the process of evolution to vaginal delivery.^(11)^ When verifying the use of the Swiss ball during labour, it was observed that such a resource can reduce pain after 20 to 90 minutes of use.^(12)^

Although the effects of maternal positioning and movement during labour are recognized^(8,11,13)^ the available evidence does not present clinical results concerning labour in induced pregnant women.

Considering the dilating effects of misoprostol induction as well as the mechanical effects of kinesiotherapy, the objective of this study was to analyse the effectiveness of kinesiotherapy in the first period of labour in pregnant women induced by misoprostol in terms of performing vaginal deliveries when compared to a usual care group.

## Methods

This is a randomised clinical trial with pregnant women in labour induced by misoprostol, which compares a group of pregnant women submitted to kinesiotherapy guidelines during the active phase of labour with a control group of pregnant women who followed the usual care of the service during the active phase of labour. The study was carried out at Lauro Wanderley University Hospital (HULW), in the city of João Pessoa, Brazil, from March to December 2021.

The following inclusion criteria were considered: pregnant women aged 18 years or older, labour induced by the drug Misoprostol (25mcg, vaginally), and single pregnancy with a live fetus. Those women diagnosed with physical and/or mental disability, fetal malformation, and women in the expulsive period of labour were excluded.

A checklist was applied by the researchers to confirm the eligibility criteria for the study. If the pregnant woman was eligible, she received information about the research and was invited to participate. Pregnant women who agreed to participate signed a Free and Informed Consent Term (TCLE)

The sample size calculation was performed using as a parameter the data from the pilot study carried out previously, in which 100% of the patients evolved to normal delivery after performing kinesiotherapy in the first period of delivery and 60% of those who did not receive kinesiotherapy evolved to vaginal delivery. For this calculation, the program Open-epi 3.0 was used. For an alpha error of 0.05 and a beta error of 0.20, a sample of 32 patients was obtained, 16 randomised to the study group and 16 randomised to the control group.

Randomization for classification into an intervention group (IG: Kinesiotherapy) or a control group (CG: Usual care) was performed according to a table of random numbers that were previously generated on a computer using the program Random Allocation Software version 1.0. Opaque and sequentially numbered envelopes were prepared, with each number, according to the randomization table, corresponding to the allocation of the pregnant woman in the study or control group. To ensure allocation confidentiality, both randomization and envelope preparation was performed by a researcher with no involvement with the research.

**Figure 1:**
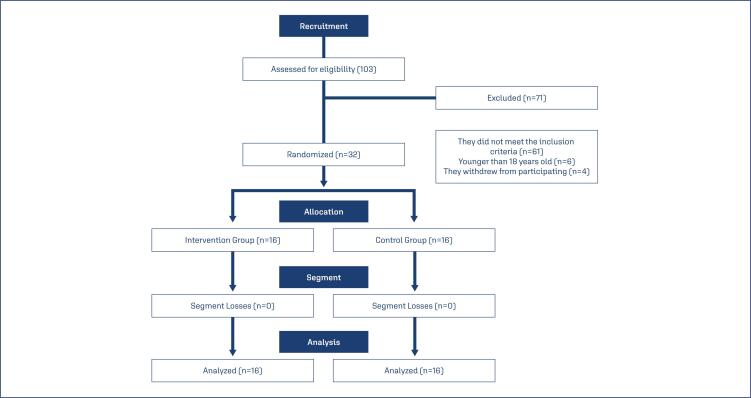
Flowchart of participant recruitment and follow-up

Patients who after randomization were allocated to the control group were treated according to usual hospital care. Those pregnant women randomised to the intervention group were followed up by a single researcher who evaluated and applied the exercises according to the protocol established by the study. Active kinesiotherapy was conducted as part of the therapeutic intervention. The protocol is available in the supplementary materials of this file (Figure 1). During the exercises, the pregnant women had free choice of when to start or stop the suggested movements. The exercises were used according to the period of dilation, fetal position, and fetal height. If there were any maternal and/or fetal complications during labour that made it impossible to carry out the interventions or the pregnant woman voluntarily decided to leave the study, the exercises would be interrupted, however, none of these situations occurred (Figure 1).

N refers to the number of participants

Pregnant women were evaluated by a single researcher after randomization and every 1 hour afterwards. The evaluation consisted of a sociodemographic questionnaire developed by the authors to characterise the sample with information on the characteristics of the induction process and labour, variables related to childbirth, maternal postpartum complications, and level of maternal pain. The primary outcome was a vaginal delivery and secondary outcomes number of misoprostol doses used, induction time, duration of the active phase and the expulsive period of labour, time of cervical dilatation, maternal pain, and the occurrence of grade 3 or 4 lacerations, and 5th minute APGAR.

To determine the level of maternal pain, the Visual Analogue Scale (VAS) was used, which varies in number from zero to 10, with zero being the total absence of pain and 10 being the extreme presence of pain. The assessment of cervical dilatation based on vaginal examination was performed by the physician on duty. To determine the time of labour, the information present in the partogram was collected. Data regarding the induction process, such as the number of doses, induction time, and use of oxytocin were collected from the induction form attached to the patient's medical record. The type of delivery and occurrence of laceration was verified at the time of delivery. The 5th-minute APGAR assessment was performed by the paediatrician on duty and was collected through the assessment.

The collected data were entered into a database of the statistical software Statistical Package for the Social Sciences version 20.0, where the statistical analysis of the results was performed, assigning a significance level of 95% (p>0.05). Descriptive statistical analysis was performed using mean, standard deviation, and confidence intervals for continuous quantitative variables and percentage for qualitative variables.

To test the hypothesis of normality of each quantitative variable, the Kolmogorov-Smirnov adherence test was used. Then, the behaviour of the quantitative variables was evaluated for the comparison of means, using the t-student test of independent samples, if normal distribution, or the Mann-Whitney test, if non-normal data distribution. The relative risk of dichotomous categorical variables with respective confidence intervals was analysed and the number needed to treat and cause benefit (NNTB) was calculated. The study was approved by the Ethics and Research Committee of the Health Sciences Center of the Federal University of Pernambuco, in accordance with the guidelines of the National Health Council (Resolution 466/2012), under registration number, *Certificado de Apresentação para Apreciação Ética* 40109320.0.0000.5208 and approval opinion number 4.451.577.

## Results

During the data collection period, 103 pregnant women were evaluated to verify eligibility. Of these, only 32 were eligible and randomised, 16 of them were allocated to the intervention group (IG) and 16 to the control group (CG). The pregnant women in the research had a mean age of 29 years (GI: 28.09 ± 6.24; CG: 30.20 ± 6.64) and had a mean gestational age of 39.2 weeks. There was no difference in terms of sociodemographic characteristics between the groups (Table 1). Likewise, there was no difference between the studied groups regarding the mean induction time; regarding the number of doses of misoprostol administered during labour, there was no statistical difference between the groups (p=0.537), however, most of the sample of pregnant women in the IG (62.5%) needed only 1 dose for the evolution of labour (Table 2).

**Table 1:** Characterization of the sample between the kinesitherapy group and the usual care group in pregnant women induced by misoprostol

		Intervention Group (n=16) n(%)	Control Group (n=16) n(%)
Marital status			
	Single	2(12,5)	5(31,2)
	Married	4(25)	3(18,8)
	Divorced	0(0)	0(0)
	Widow	0(0)	0(0)
	Consensual union	10(62,5)	8(50)
Origin			
	João Pessoa and metropolitan region	16(100)	15(93,8)
	Interior of Paraíba	0(0)	1(6,2)
	Other states	0(0)	0(0)
Education			
	Incomplete elementary school	1(6,2)	0(0)
	Completed elementary	8(50)	7(43,8)
	Incomplete high school	0(0)	1(6,2)
	Complete high school	3(18,8)	6(37,5)
	Incomplete higher education	2(12,5)	0(0)
	Complete higher education	2(12,5)	2(012,5)
Occupation			
	Housewife	2(12,5)	3(18,8)
	Others	10(62,5)	8(50)
	Absent	4(25)	5(31,2)
Parity			
	Nulliparous	7(43,8)	8(50)
	Multiparous	9(56,2)	8(50)
BMI			
	Low weight	2(12,5)	0(0)
	Adequate	3(18,8)	2(12,5)
	Overweight	6(37,5)	3(18,8)
	Obesity	4(25)	6(37,5)
	Absent	1(6,2)	5(31,2)

**Table 2:** Comparison of the induction process between the kinesiotherapy group and the usual care group in misoprostol-induced pregnant women (Number of misoprostol doses and total induction time)

Induction time	Intervention Group (n=16)	Control Group (n=16)	p-value	Mean Differences	CI 95%
	Mean ± SD	Mean ± SD			
Time (Hours)	11:27 ± 12:07	16:46 ± 14:40	0,164	-5,300	(-15,0 - 4,4)
**Doses of misoprostol administered**	**n(%)**	**n(%)**			
1 dose	10(62,5)	5(31,2)			
2 - 3 doses	4(25)	6(37,5)			
4 doses	1(6,2)	2(12,5)			
5 - 7 doses	1(6,2)	2(12,5)			
8 doses	0(0)	1(6,2)			

* *p* = Mann-Whitney

When analysing the outcome duration of the active phase of labour, measured in hours (GI: 6.37 ± 4.03; CG: 9.16 ± 16.4) and duration of the second stage (GI: 0.32 ± 0.40; CG: 0.48 ± 1.13) there was no difference between the studied groups (Table 3). Regarding the analysis of the time between the beginning of dilation and complete dilation, measured in hours (GI: 20.22 ± 10.25; CG: 21.6 ± 14.13), there was no statistical difference between the means (p=0.92).

**Table 3:** kinesiotherapy group and the usual care group in misoprostol-induced pregnant women

Time	Intervention Group	Control Group	p-value	Difference between the means	CI 95%
	Mean ± SD	Mean ± SD			
Duration of active phase (hours)	6:37 ± 4:03	9:16 ± 16:4	0,440	-2,641	(-11,0 - 5,7)
Duration of expulsive period (hours)	0,32 ± 0,40	0,48 ± 1,13	0,340	-0,235	(-1,0 -0,5)

* *p* = Mann-Whitney

It was not possible to observe a difference in the level of maternal pain at the beginning of the active phase (5-6 cm of dilation), in the middle of the active phase (7-8 cm of dilation), and at the end of the active phase (9-10 cm of dilation) (Table 4). As well as in the performance of instrumental delivery, presence of laceration, and degree of laceration (Table 5). However, there was a difference regarding the mode of delivery, where most vaginal deliveries were performed in the intervention group (RR=11.6; 95%CI [1.2-110.9] p= 0.016), with a number necessary to treat an individual to benefit (NNTB) of 2 (95% CI 1 to 5) (Table 5).

**Table 4:** Analysis of pain using the Visual Analog Scale (VAS) between the kinesiotherapy group and the usual care group in misoprostol-induced pregnant women

Cervical dilation (cm)	Intervention group	Control group	p-value	Difference between the means	CI 95%
	Mean ± SD	Mean± SD			
Beginning (5-6 cm)	6,1 ± 1,9	7,06 ± 3,27	0,276	-0,875	(-2,8 - 1,0)
Middle (7-8 cm)	7,68 ± 1,19	8 ± 2,68	0,172	-0,313	(-1,8 - 1,1)
End (9-10cm)	9 ± 1,41	8,56 ± 2,78	0,595	0,438	(-1,1 - 2,0)

* *p* = Mann-Whitney

**Table 5:** Comparison of the mode of delivery, instrumental delivery, and lacerations between the physiotherapy group and the usual care group induced by misoprostol

		Intervention group (n=16)	Control group (n=16)	RR	IC95%	p-value^a^
		n(%)	n(%)			
Delivery route						
	Vaginal	15(93,8)	9(56,2)	11,6	(1,2-110,9)	0,016
	Caesarean section	1(6,2)	7(43,8)			
Instrumental delivery b						
	Yes	1(6,7)	0(0)	0,9	(0,8-1,0)	0,453
	No	14(93,3)	9(100)			
Presence of laceration b						
	Yes	11(73,3)	5(55,6)	2,2	(0,3-12,5)	0,381
	No	4(26,7)	4(44,4)			
Degree of laceration b						
	1 and 2	9(84,6)	5(100)	0,8	(0,6-1,06)	0,324
	3 or 4	2(15,3)	0(0)			

* RR = Relative Risk; CI = Confidence Interval;

a *p-value* = Mann-Whitney;

b The missing numbers refer to cesarean deliveries

## Discussion

The implementation of therapeutic exercises is an important intervention resource in delivery rooms, however, its effectiveness is still little explored, and this study is a pioneer in its analysis combined with the use of the inducing drug misoprostol. According to our observations, it is possible to affirm that a significant difference was observed in the effectiveness of kinesiotherapy regarding the mode of delivery, proving to be effective in the evolution of the outcome of the vaginal delivery type. Such a difference was not evidenced in the other variables studied.

The success of misoprostol induction is directly related to the number of doses administered,^(5)^ however, the costs of maternal hospitalisation in induced labour are still on average 16.9% higher compared to the labour of spontaneous delivery.^(14)^ Therefore, it is of fundamental importance to adopt measures that seek to minimise this financial impact on the health system. From this perspective, this study did not find a statistical association between kinesiotherapy and the number of doses of misoprostol, however, the administration of only one tablet was necessary for the majority (62.5%) of pregnant women in the GI to progress to labour, which has a benefit both financially and clinically.

In isolation, misoprostol induction, although widely used, does not guarantee the evolution to vaginal delivery. Studies show a high number of caesarean sections in induced women,^(5,6)^ this type of procedure delivery is related to high costs, which can be higher by up to 32% compared to vaginal delivery, resulting from procedural expenses and longer hospital stays.^(15)^ Given this, our research has shown that when associated with kinesiotherapy, misoprostol induction results in a vastly greater number of vaginal deliveries.

During kinesiotherapy, the exercises performed were executed in the orthostatic position, which is proven to be beneficial in reducing the time of the active phase of labour,^(8)^ nevertheless, the implication of this result in induced women was not yet known. In the current study, no statistical difference was found between the groups analysed regarding the duration of the active phase of labour and the expulsive period.

The presence of a physical therapist accompanying the pregnant woman can have an important effect on maternal satisfaction, as it leads to a lower occurrence of negative feelings about the birth experience.^(16)^

In the current study, within the group of induced pregnant women, the level of pain was investigated, regardless, no difference was identified between the groups studied.

Monitoring during labour can interfere with better maternal and fetal outcomes, such as more adequate five-minute APGAR scores.^(16)^ In the groups observed in our study, it was possible to verify that all births had APGAR between 6 and 10 in the 5th minute.

The therapeutic exercise program is a fundamental and unique component of the physical therapist that applies concepts of physiology, anatomy, and kinesiology in which, combined, they culminate in functional results for patients,^(17,18)^ results observed in current research for the effectiveness in increasing vaginal deliveries in GI. Several studies have already shown the beneficial influence of the presence of a physical therapist during labour,^(11,17,19)^ however, the presence of this professional in maternity hospitals is still not something concrete in several countries. This makes it necessary to implement policies to incorporate the physical therapist in the multidisciplinary team for comprehensive care in maternity hospitals.

This study has some limitations that should be considered when interpreting the results. First, it is a single-center clinical trial, which may limit the generalizability of the findings to other populations and clinical settings. In addition, despite the randomization, it was not possible to completely blind the participants, and the professionals involved in the intervention, which may have introduced performance and detection bias.

Another limitation concerns the sample size, which, although calculated based on previous estimates, may not have been sufficient to detect significant differences in some secondary outcomes.

## Conclusion

The findings of this study indicate that after the application of kinesiotherapy, during the active phase of labour in pregnant women induced by misoprostol, the number of vaginal deliveries increases. No statistical differences were found in the other variables studied, such as induction time, duration of the active phase and expulsive period, pain level, presence of laceration, Apgar, and fetal weight.
